# Transcriptional Regulation of *Vih* by Oct4 and Sox9 in *Scylla paramamosain*

**DOI:** 10.3389/fendo.2020.00650

**Published:** 2020-10-15

**Authors:** Jiaqian Liao, Ziping Zhang, Xiwei Jia, Zhihua Zou, Keying Liang, Yilei Wang

**Affiliations:** ^1^Fujian Engineering Research Center of Aquatic Breeding and Healthy Aquaculture, Fisheries College, Jimei University, Xiamen, China; ^2^Key Laboratory of Healthy Mariculture for the East China Sea, Ministry of Agriculture and Rural Affairs, Xiamen, China; ^3^College of Animal Science, Fujian Agriculture and Forestry University, Fuzhou, China; ^4^Key Laboratory of Marine Biotechnology of Fujian Province, Institute of Oceanology, Fujian Agriculture and Forestry University, Fuzhou, China

**Keywords:** transcriptional regulation, *Vih*, Oct4, Sox9, *Scylla paramamosain*

## Abstract

Mud crab (*Scylla paramamosain*) is one of the most economically-important marine crabs in China. However, research on mechanisms of reproductive regulation is not sufficient. Vitellogenesis-inhibiting hormone (VIH) is a member of the crustacean hyperglycemia hormones (CHH) family, which plays an essential role in the regulation of gonadal development and maturation in crustaceans, and current studies on the regulation of *Vih* transcription in crabs are relatively rare. Our previous studies on the transcriptional regulation of mud crab *Vih* (*SpVih*) have proved that the binding site of Oct4/Sox9 transcription factor may be the key region for positively regulating the expression of *SpVih*. In this study, the electrophoretic mobility shift assay (EMSA) experiment confirmed that the nuclear protein extracted from the eyestalk could bind to the key region of *SpVih* promoter, and these specific bindings were dependent on the presence of Oct4/Sox9 binding sites. Two specific binding complex bands were detected in the supershift group of EMSA supershift experiments by Oct4 and Sox9 antibodies, further confirming the specific recognition of these two transcription factors on the key regulatory region of *SpVih*. *In vitro, Oct4* and *Sox9* gene overexpression vectors and *SpVih* core promoter fragment vector were constructed and co-transfected into HEK293T cells. As a result, *SpVih* activity increased with the concentration of transcription factors. *In vivo*, when *Oct4* and *Sox9* dsRNA were injected into the eyestalks of mud crab, respectively, the expression level of *SpVih* decreased significantly after interference with *Oct4* or *Sox9*, and the expression level of *SpVtg* in the ovary and hepatopancreatic increased. Both *in vitro* and *in vivo* experiments showed that Oct4 and Sox9 had a positive regulatory effect on *SpVih*. The GST pull-down experiment was carried out by purified Oct4 and Sox9 proteins, and the results showed that there was an interaction between them. It was speculated that they regulated the expression of *SpVih* through the interaction.

## Introduction

Vitellogenesis-inhibiting hormone (VIH), also known as gonad-inhibiting hormone (GIH), is a member of the crustacean hyperglycemia hormone (CHH) family secreted from the X-organ sinus gland complex (XO-SG) of eyestalk (1). VIH was first isolated from the eyestalk of *Homarus americanus*, and it has been described as a vitellogenesis inhibitor because it exerts a significant negative regulatory effect during gonad maturation (2, 3). Thus far, eyestalk ablation is commonly practiced in shrimp and crab to induce ovarian maturation in captivity. Nevertheless, there are a lot of drawbacks to this approach, such as increased parental mortality, lower egg quality, and lower hatching rate (4), etc. In order to solve this problem, many scholars began to try to find some reliable alternative methods. For example, Marins's group first used RNAi technology to silence GIH transcripts to develop an alternative approach to eyestalk ablation in captive shrimp *Litopenaeus vannamei* (5). Devaraj et al. (6) first used molecular signal intervention, a less invasive method than traditional eyestalk ablation, to suppress VIH expression via the MEK pathway to induce ovarian maturation in female *Penaeus monodon*. Treerattrakool et al. (7) reported the preparation of monoclonal antibody specific to Pem-GIH (anti-GIH mAb) and implication of antibody neutralization on the induction of ovarian maturation in *P. monodon*.

Mud crab (*Scylla paramamosain*) is one of the most important marine crabs in China, but the seedlings used in aquaculture operations are mainly obtained from wild sources (8). The shortage of seedling has severely hindered the development of mud crab aquaculture. In order to address the above problem, it is urgent to study the molecular regulation mechanism of gonadal development and maturation in mud crab. Transcription factors are essential molecules that control gene expression. They can activate or repress gene transcription through binding to specific sites of gene promoters, which are essential for a series of crucial cellular processes. The study of transcription factors controlling the expression of *SpVih* may also be one of the methods to develop alternative eyestalk ablation in the future. In the preliminary study on the transcriptional regulation of the *SpVih* gene in our laboratory, we found the key regulatory element in *SpVih* promoter and the key transcription factors, Oct4 and Sox9, that may regulate the expression of *SpVih* (9). Based on this research, this study will explore the regulation of *SpVih* expression by these two transcription factors.

Transcription factor Oct4 belongs to the Pou protein family, and its main function is to form and maintain pluripotent stem cells. In many vertebrates, the *Oct4* gene is highly or specifically expressed in early embryos, proving that it is indispensable for the early development of embryos. In terms of gonad development, Oct4 is highly expressed in primordial germ cells and mature gonads in a variety of vertebrates (10, 11). As a transcription factor, Oct4 participates in the regulation of various target genes. Jen et al. (12) found that Oct4 can activate the transcription of MALAT1, thereby promoting cell proliferation and movement. Wu et al. (13) found that the transcription factor Oct4 regulates the transcriptional activity of Dnmt1. In the mud crab, it was found in our previous studies that *Oct4* mRNA was highly expressed in the mature ovaries, and its protein could also be detected in the ovaries. Meanwhile, both Oct4 mRNA and protein were highly expressed in the eyestalk of female crabs (14).

Sox9 belongs to the SoxE subfamily of the Sox gene family. It is a critical factor in the sex determination and sex differentiation process of many animals. In mammals, it is a key gene in the testicular determination pathway (15, 16), similar effects have been found in some amphibians and reptiles (17, 18). Sox9 was found to be expressed at high levels or specifically in the testis in many bony fishes, such as *Pelteobagrus fulvidraco* (19), *Betta splendens* (20), *Pelteobagrus fulvidraco* (21), *Acipenser baerii Brandt* (22), and *Oncorhynchus mykiss* (23), etc. In addition to being involved in testis development, Sox9 has been found to be expressed in oocytes. It carries an additional function in the posttranscriptional processes in some amphibians such as *Pleurodeles waltl, Xenopus laevis*, and *X. tropicalis* (24). Researches on invertebrate *Bombyx mori* (25), *Drosophila melanogaster* (26), and *Apis florea* (27) have also found that homologous genes of Sox9 are indispensable for their sex differentiation and gonadal development. As a transcription factor, Sox9 is also involved in the regulation of multiple target genes. In mice, embryo knockout and cell transfection experiments confirmed that Sox9 and SF1 could upregulate the expression of *Cyp26b1* during gonadal development to maintain the development of germ cells in males (28). Minerva et al. (29) found that Sox9 can also enhance the activity of the *Catsper1* promoter, together with the transcription factor Sox5. In the tissues of mature mud crab, Sox9 was only expressed in the gonads, eyestalk, and cerebral ganglia, suggesting that it may play a role in the development of gonads (14).

## Materials and Methods

### Animal and Tissue Collection

All mud crabs with good vitality were purchased from the Jimei market, Xiamen city, Fujian province. The eyestalk of immature female crabs weighing about 100 g was used to isolate nucleoproteins. Female crabs weighing about 250 g were temporarily kept in the seawater field of Jimei University for a week before the RNA interference experiment. After the experiment, the eyestalks, gonads, and hepatopancreas of the crabs were sampled and frozen immediately in liquid nitrogen, then stored at −80°C for total RNA extraction. All animal experiments were conducted following the regulations of the Guide for Care and Use of Laboratory Animals and were approved by the Animal Ethics Committee of Jimei University.

### Electrophoretic Mobility Shift Assay (EMSA)

In order to detect whether there is a specific binding site for Oct4 or Sox9 at the core regulatory region of *SpVih*, EMSA experiments were performed. Briefly, according to the manufacturer's protocol for the LightShift Chemiluminescent EMSA Kit (Thermo Scientific, Waltham, MA, USA), biotin-labeled and unlabeled double-stranded DNA probes containing intact core recognition element (CRE) for Oct4 and Sox9 were prepared. For competitive analysis, the mutant probe was synthesized. Nuclear proteins were extracted from the eyestalk of immature female crabs using NE-PER Nuclear and Cytoplasmic Extraction Reagent (Thermo Scientific). In the competition experiment, we set up a 100-fold amount of mutation probe group and a 10- and 100-fold amount of unlabeled probe group, respectively. Supershift group added Oct4 or Sox9 antibodies (Abcam, Cambridge, UK, ab19857 and ab3697). Protein-DNA complexes were separated on 6% polyacrylamide gels in 1× TBE via electrophoresis at 100 V for 1 h and transferred onto nylon membranes. Chemiluminescence detection was carried out according to the Chemiluminescent Nucleic Acid Detection Module kit (Thermo Scientific).

### Plasmid Construction, Cell Culture, Transfection, and Dual-Luciferase Reporter Assay

For overexpression studies, the *SpVih* core promoter fragment was amplified by PCR, and then cloned into the Kpn I-Xho I site of the pGL3-basic vector. The full-length ORF of Oct4 and Sox9 was cloned into the pcDNA3.1 vector, respectively. All plasmid constructs were verified by sequencing analysis. The primers with the restriction enzyme cutting sites were listed in Table 1.

**Table 1:** Primers used in this study.

**Primer name**	**Sequence (5^**′**^−3^**′**^)**	**Usage**
*Sp*TF-wt-F	GGCATGGTTCCGGGCAGAAATATAAAGTGCATGAGATTCTGGTGAAGGAAGGCTTAAGTA	EMSA
*Sp*TF-wt-R	TACTTAAGCCTTCCTTCACCAGAATCTCATGCACTTTATATTTCTGCCCGGAACCATGCC	EMSA
*Sp*TF-mut-F	GGCATGGAACCGGGCAGAAATATAAAGTGCATGAGATTCTGGTGAAGGAAGGCTTAAGTA	EMSA
*Sp*TF-mut-R	TACTTAAGCCTTCCTTCACCAGAATCTCATGCACTTTATATTTCTGCCCGGTTCCATGCC	EMSA
*Sp*Vih-4-F	cggGGTACCTGGTTCCGGGCAGAAATATAAAGT	Cell transfection
*Sp*Vih-4-R	atttGCGGCCGCGACGAAACTCAATGAACAC	Cell transfection
*Sp*Sox9*-*ORF-F	cggGGTACCAACTCCATCACCAACTACATTCATT	Cell transfection
*Sp*Sox9-ORF-R	atttGCGGCCGCCCGACTGCGGTGTAGAGGAG	Cell transfection
*Sp*Oct4-ORF-F	cggGGTACCATGGCTACAACAACTTACATCGCAT	Cell transfection
*SpOct4-ORF-R*	atttGCGGCCGCGGTTGAGCGACCATCAGTGGGAGAC	Cell transfection
Oct4-RNAi-F	AGACCACCATCTGCAGGTTC	dsRNA synthesis
Oct4-RNAi-R	ACACGCACCACCTCTTTCTC	dsRNA synthesis
Sox9-RNAi-F	AGGAATGGGGTCACGTTGG	dsRNA synthesis
Sox9-RnAi-R	ATCGGTTCTCGCCTGTTGC	dsRNA synthesis
EGFP-RNAi-F	GGTGAACTTCAAGATCCGCC	dsRNA synthesis
EGFP-RNAi-R	CTTGTACAGCTCGTCCATGC	dsRNA synthesis
*Sp*Oct4-RT-F	AGACCACCATCTGCAGGTTC	qRT-PCR
*Sp*Oct4-RT-R	TCCGCTTTTTACGTTTCCTG	qRT-PCR
*Sp*Sox9-RT-F	TCCACGTGAAGAGGCCAATG	qRT-PCR
*Sp*Sox9-RT-R	CATGCCCCTCCATATCCTGC	qRT-PCR
*Sp*Vih-RT-F	AGGAGGAACTGCTTCTACAACGAGG	qRT-PCR
*Sp*Vih-RT-R	GAGTGAATAATGTGAGATGTGGCTA	qRT-PCR
18S-rRNA-RT-F	ATGATAGGGATTGGGGTTTGC	qRT-PCR
18S-rRNA-RT-R	AGAGTGCCAGTCCGAAGG	qRT-PCR
*Sp*Vtg-RT-F	CGTACCGGACATCTTCCGAG	qRT-PCR
*Sp*Vtg-RT-R	ACGAGCCCACTACAGAGACT	qRT-PCR
*Sp*Oct4-F	cgcGGATCCATGGCTACAACAACTTACATCGCAT	Prokaryotic expression
*Sp*Oct4-R	ccgCTCGAGGGTTGAGCGACCATCAGTGGGAGAC	Prokaryotic expression
*Sp*Sox9-F	ccgCTCGAGAACTCCATCACCAACTACATTCATT	Prokaryotic expression
*Sp*Sox9-R	cccAAGCTTCCGACTGCGGTGTAGAGGAG	Prokaryotic expression

*The underline indicates the primer cleavage site, lowercase English letters indicate protective bases*.

HEK293T cell line was cultured in the DMEM medium supplemented with 10% FBS (fetal bovine serum, Gibco, Waltham, USA) and 1% (v/v) antibiotic (penicillin/streptomycin, Gibco, Waltham, USA) at 37°C in a humidified incubator with 5% CO_2_. The medium was changed upon the state of cells.

Transfection of plasmids in HEK 293FT cells were performed using Lipofectamine 2000 (Invitrogen, Carlsbad, CA, USA) according to manufacturer's instructions. pRL-TK plasmid (10 ng) was used as an internal control. The transfection reagent and plasmids (Oct4 and Sox9 at 50 ng, 100 and 200 ng, respectively, or 25 ng Oct4 + 25 ng Sox9, 50 ng Oct4 + 50 ng Sox9, and 100 ng Oct4 + 100 ng Sox9) were mixed and co-transfected into the HEK293T cell lines. At 3–6 h post-transfection, the transfection mixture was replaced with DMEM containing 10% FBS. The pEGFP-N1 plasmid was employed as a positive control. After continuing culture for 24 h, the cells were harvested. The firefly and renilla luciferase activities were measured by the Dual-Luciferase Reporter Assay System (Promega, Madison, USA) according to the manufacturer's protocol. The promoter activity was assessed by the average of firefly luciferase activity normalized as a ratio to the renilla luciferase activity. Each experiment was conducted for three independent samples in triplicate.

### RNA Interference

In *in vivo* experiments, the 286 and 275 bp-length ORF of Oct4 and Sox9, respectively, were chosen as the templates for synthesizing gene-specific dsRNA. Enhanced green fluorescent protein (EGFP) gene was used as a negative control group, and its 229 bp-length ORF was selected as a template for synthesizing EGFP dsRNA. These dsRNAs were synthesized from the linearized template by using the T7 RiboMAXTM Express RNAi System (Promega). These dsRNAs were uniformly diluted to 1 μg/μL, and each crab was injected with 10 μL of dsRNA on the base of each eyestalk. The blank control group was injected with the same volume of stroke-physiological saline solution (SPSS). After continuous injection for 3 days, the eyestalks, gonads, and hepatopancreas were dissected on the fourth day and placed in RNA later and then transferred to −20°C overnight for RNA extraction.

### RNA Extraction and Reverse Transcription

Total RNA was extracted from different tissues using Total RNA Extraction Kit (Promega, Shanghai, China) according to the manufacturer's protocol. Total RNA quality was assessed by agarose gel electrophoresis and Nanodrop 2000 (Thermo Scientific). The complementary DNA (cDNA) was synthesized in a 20 μL reaction system including 1 μg total RNA (previously treated with DNase I), 2 μL random primers (10 mM), 4 μL 5× First-strand Buffer, 1 μL dNTP mix (10 mM), and 1 μL M-MLV reverse transcriptase (200 U/μL) (Promega, Shanghai, China). The synthesized cDNA was diluted and stored at −20°C until use.

### Quantitative Real-Time PCR (qRT-PCR) Analysis

qRT-PCR was carried out in a LightCycler480 Roche Realtime Thermal Cycler (Mannheim, Baden-wurttemberg, Germany) in accordance with the manual with a 20 μL reaction volume containing 9 μL of 1:100 diluted original cDNA, 10 μL SYBR Green Master Mix (Promega, USA), 0.5 μL of the forward primer and 0.5 μL of reverse primer (10 mM). Primer sequences are shown in Table 1. *18s rRNA* was used as the reference gene. The cycling conditions for PCR reaction were set as follows: 1 min at 95°C, followed by 40 cycles at 95°C for 15 s, 59°C for 1 min. Quantitative measurements were performed using the ΔΔCt method. Each sample had at least 4 biological replicates. Statistical analysis was performed by one-way analysis of variance (one-way ANOVA) using Statistical Package for the Social Sciences 20.0 (SPSS 20.0) software (IBM Corporation, New York, NY, USA). The statistically significant differences were shown at *p* < 0.05; the most significant differences were shown at *p* < 0.01.

### Recombinant Expression and Purification

According to the full-length cDNA sequences of Oct4 and Sox9, two primer pairs were designed to amplify the sequences that encode their corresponding fragments, respectively (Table 1). The amplified fragment of Oct4 was digested by BamHI and XhoI, and then inserted into a pGEX-4T-1 vector. Meanwhile, the Sox9 fragment was ligated into a pET30a expression vector after being cut by NdeI and HindIII. Two recombinant expression vectors, namely pGEX-4T-Oct4, pET30a-Sox9, were transformed into competent *Escherichia coli* Rosetta (DE3) host cells. Isopropyl-b-D-thio-galactoside (IPTG) was added to induce protein expression. The recombinant Oct4 protein with a glutathione S-transferase (GST) tag was purified using glutathione Sepharose 4B chromatography (Detai Biologics, Nanjing, China) according to the manufacturer's instructions. Sox9 protein with an His-tag was harvested by His Bind resin chromatography (Detai Biologics) according to the manufacturer's instructions.

### GST Pull-Down Assay

Approximately 15 μg of purified recombinant Oct4 was incubated with glutathione sepharose for 1 h at 4°C and then washed with binding buffer (1× PBS, pH = 7.4). Afterward, ~15 μg of purified recombinant Sox9 was added and then incubated for 1 h at 4°C. After being washed thoroughly with binding buffer (1× PBS, pH = 7.4), the proteins were eluted with elution buffer (30 mM GSH, 50 mM Tris, 0.1% Triton X-100) and then finally analyzed by SDS-PAGE and Western blot. GST protein was used as the negative control in this assay.

## Results

### Confirmation of Oct4 and Sox9 Binding to *SpVih* Promoter by EMSA

**Figure 1:**
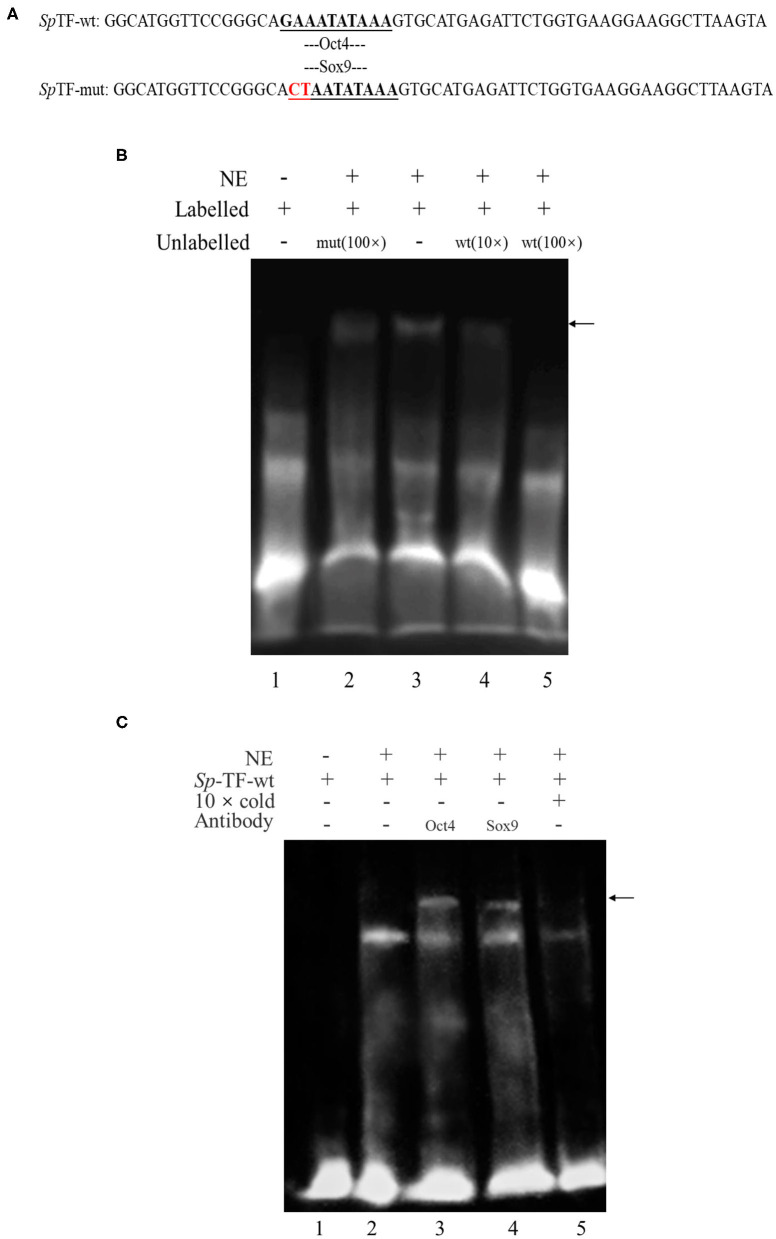
Electrophoretic mobility shift assay (EMSA) analysis of the binding of Oct4 and Sox9 to specific CRE in the *SpVih* promoter. **(A)** The sequence of the wild-type probe and the mutant probe. **(B)** EMSA. Biotin-labeled and unlabeled double-stranded DNA probes containing complete CRE of Oct4 and Sox9 as well as mutated probes that mutated the binding sites of Oct4 and Sox9 were prepared. Lane 1 was the free probe. The *Sp*TF-wt could combine with the eyestalk nuclear extract to form a DNA-protein complex (lane 3). The formation of the complex was not affected when 100-fold of unlabeled *Sp*TF-mut and *Sp*TF-wt co-existed (lane 2). When the cold probe was added, the binding band will be competitively bound by the cold probe (lane 4-5). **(C)** EMSA supershift. Biotin-labeled and unlabeled double-stranded DNA probes containing Oct4 and Sox9 intact CRE were prepared. Lane 1 was the free probe. The *Sp*TF-wt could combine with the eyestalk nuclear extract to form a DNA-protein complex (lane 2). The unlabeled intact probe could compete for the binding of Oct4 and Sox9 to the labeled intact probe (lane 5). After Oct4 and Sox9 antibodies were added, a supershift band appeared (lane 3-4), and the black arrow indicates the supershift band. NE, eyestalk nuclear extract.

To determine whether the transcription factor Oct4/Sox9 can recognize and bind to the corresponding site of *SpVih* promoter, the EMSA experiment was conducted after incubation with eyestalk nuclear extract and probe. The results showed that the wild-type probe *Sp*TF-wt (Figure 1A) could combine with the eyestalk nuclear extract to form a DNA-protein complex (Figure 1B, lane 3, arrow marked). The formation of this complex can be competitively bound by cold probe (wild type unlabeled *Sp*TF-wt). The binding band became shallow when it combined with 10-fold cold probe. When the amount of cold probe was 100-fold, the DNA-protein complex binding band is entirely undetectable (Figure 1B, lane 5), indicating that this complex is specific to *Sp*TF-wt. When 100-fold unlabeled *Sp*TF-mut (mutated the binding site of Oct4 and Sox9) and *Sp*TF-wt coexisted, the complex formation was not affected, indicating that the mutated probe *Sp*TF-mut did not participate in competitive binding.

To confirm the Oct4 and Sox9 binding to the promoter of *SpVih*, EMSA supershift was carried out using both biotin-labeled and unlabeled DNA probes containing intact CRE for Oct4 and Sox9. EMSA supershift with crab eyestalk nuclear extract demonstrated a DNA-protein complex formation with a biotin-labeled probe containing Oct4 and Sox9 motifs (Figure 1C, lane 2). Further, Oct4 and Sox9 antibody's addition resulted in the supershift of protein–DNA complex (Figure 1C lanes 3–4, arrow marked).

### Transcription Regulation of *SpVih* by Oct4 and Sox9

**Figure 2:**
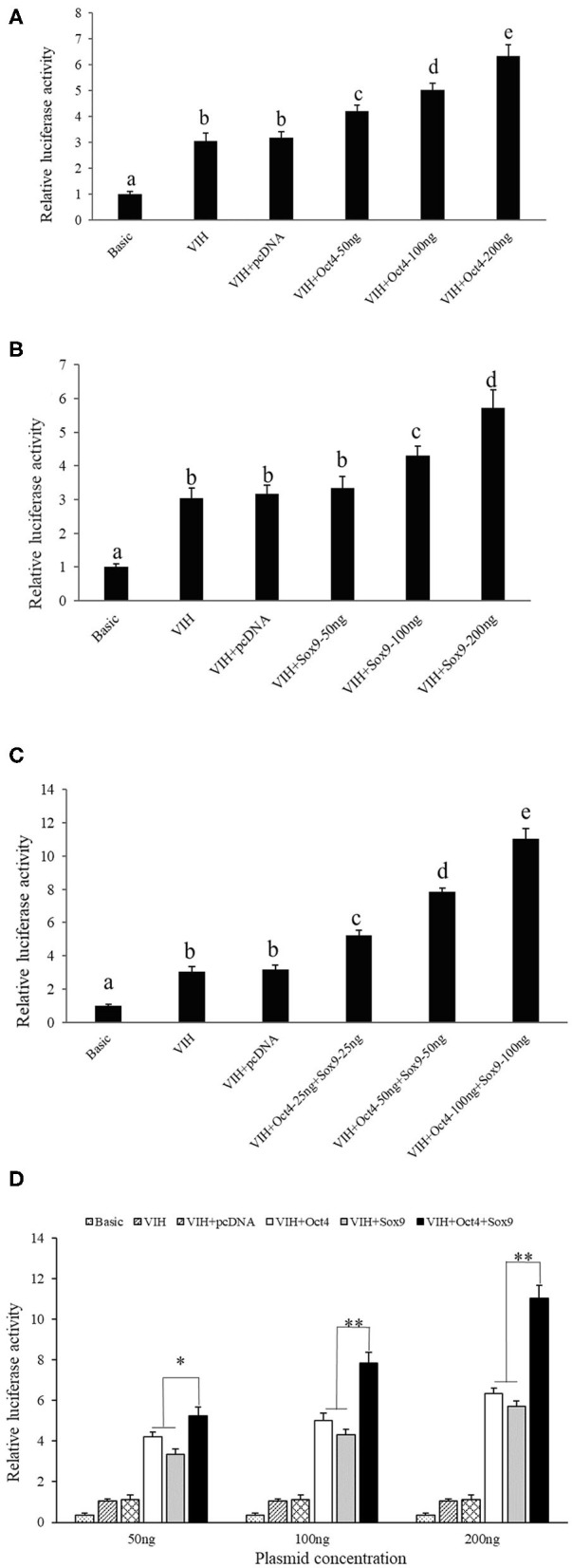
Overexpression of transcription factors can promote the activity of *SpVih* core promoter in HEK293T cell. **(A)** Effect of *Oct4* gene overexpression on *SpVih* core promoter activity. **(B)** Effect of *Sox9* gene overexpression on *SpVih* core promoter activity. **(C)** Effect of the co-overexpression of *Sox9* and *Oct4* genes on *SpVih* core promoter activity. **(D)** Effect of *Sox9* or/and *Oct4* overexpression plasmids, respectively, at different concentrations on the activity of *SpVih* core promoter. Bar with different letters indicates significant differences (*P* < 0.05). * Represents significant difference (*P* < 0.05), ** represents extremely significant difference (*P* < 0.01).

Different concentrations of Oct4 and Sox9 overexpression plasmids were co-transfected with the *SpVih* core promoter-reporter plasmid *Sp*Vih-4 into the HEK293T cell line, respectively. PGL3-basic was used as a negative control, while pRL-TK was used as the reference vector. The cells at 24 h after transfection were collected to detect reporter gene carrier activity. The results showed that the addition of either 50 ng Oct4 overexpressed plasmid or 100 ng Sox9 overexpressed plasmid could significantly promote the activity of *SpVih*, and the *SpVih* activity increased with the increment of the overexpressed plasmid concentration (Figures 2A,B). When HEK293T cells were co-transfected with two overexpression plasmids and *SpVih* fragment reporter plasmid at the same time, the activity of *SpVih* increased significantly. It showed a dose-effect with the concentration of transcription factors (Figure 2C). Compared with a single transcription factor overexpression plasmid group, co-transfection with two overexpression plasmids can significantly upregulate the activity of *Sp*Vih (Figure 2D).

### Effect of RNA Interference Experiment on *SpVih*

**Figure 3:**
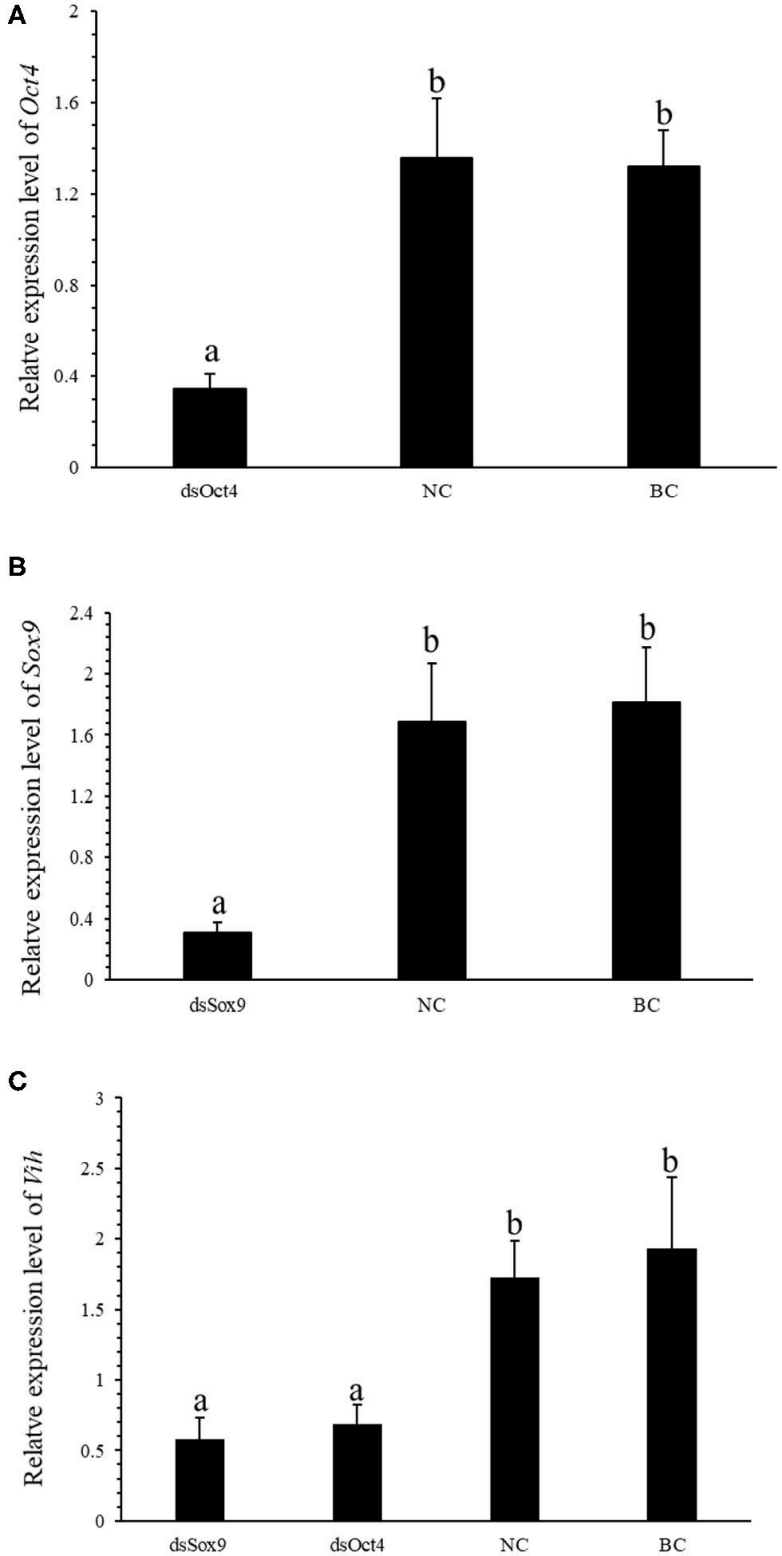
*Oct4* or *Sox9* RNA interference reduces the expression level of *SpVih*. **(A)** Relative expression of *Oct4* in the eyestalk after injection of dsOct4, dsEGFP, and SPSS. **(B)** Relative expression of *Sox9* in the eyestalk after injection of dsOct4, dsEGFP, and SPSS. **(C)** The relative expression of *SpVih* in the eyestalk after injection of dsSox9, dsOct4, dsEGFP, and SPSS. NC, negative control group; BC, blank control group; Bar with different letters indicates significant differences, *P* < 0.05.

To further verify the regulation of *SpVih* by Oct4 and Sox9 in cell experiments, we designed an *in vivo* RNA interference experiment. After 3 consecutive days of dsOct4 and dsSox9 injections, respectively, compared with the control group, the relative expression of *Oct4* or *Sox9* in the eyestalk of the experimental groups was significantly reduced (Figures 3A,B), indicating that the experiment successfully interfered with the expression of Oct4 or Sox9 in the crabs. Compared with the blank control group and the negative control group, the expression of *SpVih* of the eyestalk in the experimental group injected with dsOct4 or dsSox9 was significantly decreased (Figure 3C), indicating that interference with Oct4 or Sox9 can inhibit *SpVih* expression in the crab.

### Effect of RNA Interference Experiment on *SpVtg*

**Figure 4:**
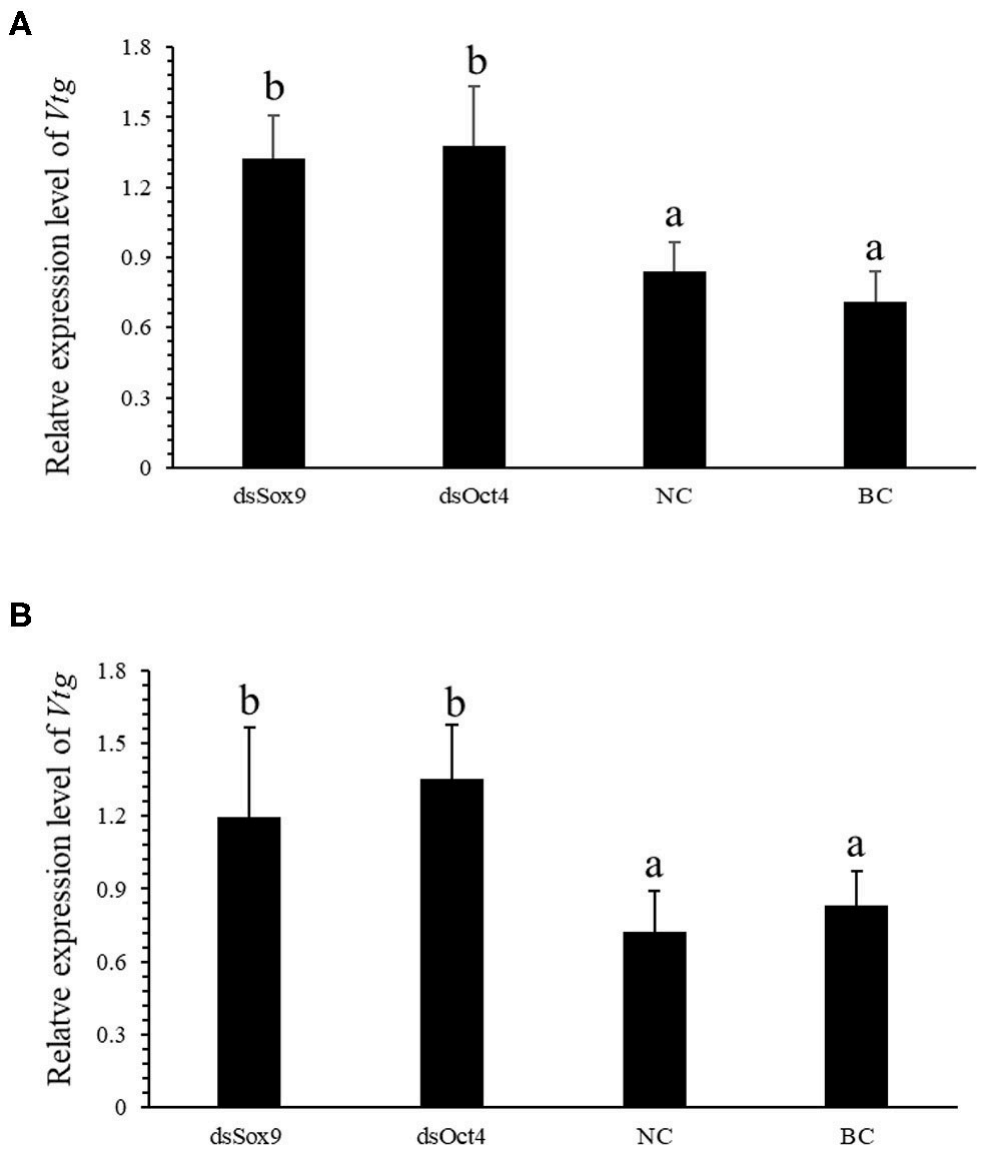
*Oct4* or *Sox9* RNA interference promotes the expression level of *SpVtg*. **(A)** Relative expression of *SpVtg* in the ovary after injection of dsSox9, dsOct4, dsEGFP, and SPSS. **(B)** The relative expression of *SpVtg* in hepatopancreas after injection of dsSox9, dsOct4, dsEGFP, and SPSS. NC, negative control group; BC, blank control group; Bar with different letters indicates significant differences, *P* < 0.05.

Vitellogenin (Vtg) is the precursor of Vitellin (Vn or Vt) and is the main component in the yolk of oviparous animals. VIH in crustaceans can inhibit the production of Vtg and regulate ovarian development and maturation. The qRT-PCR experiment was conducted to investigate the expression of *SpVih* in the eyestalk and the expression of *SpVtg* in ovary and hepatopancreas after the injection with dsOct4 and dsSox9. The experimental results showed that, compared with the blank control group and the negative control group, when dsOct4 and dsSox9 were injected into the eyestalk of the crab, the expression of *SpVih* in the eyestalk decreased significantly while the relative expression of *SpVtg* in the ovary and hepatopancreas had a significant rise (Figure 4).

### Recombinant Expression and Purification

**Figure 5:**
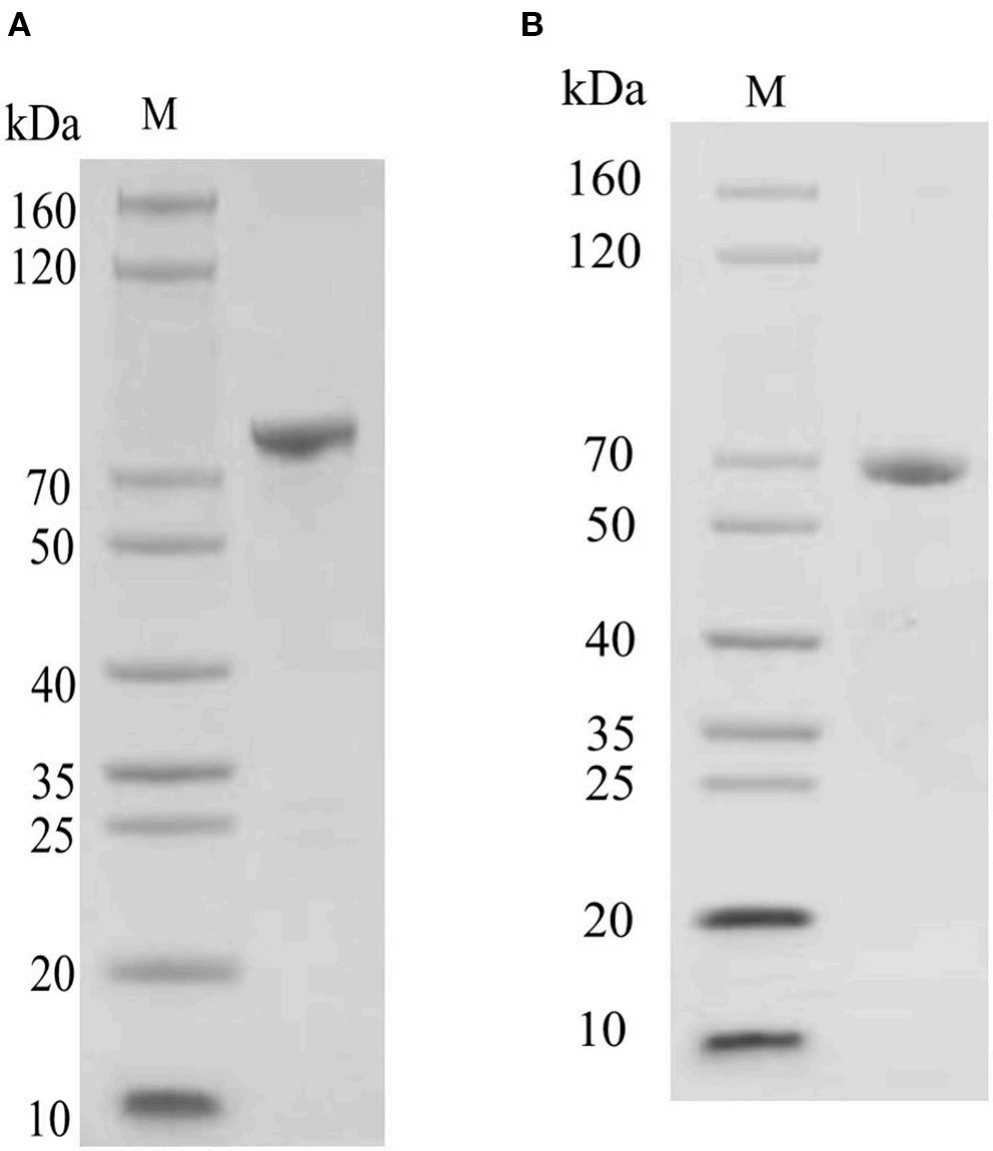
SDS-PAGE detection of recombinant protein. **(A)** SDS-PAGE detection of pGEX-4T-Oct4 protein. **(B)** SDS-PAGE detection of pET30a-Sox9 protein. M, Marker.

Oct4 was expressed as a soluble protein after IPTG induction. It was conveniently purified by glutathione sepharose 4B chromatography. The purified protein comprised of an ~44 kDa Oct4 protein and a nearly 26 kDa GST tag expressed by the plasmid pGEX4T1, which was roughly consistent with the size (about 70 kDa) of the unique band in the purified protein lane (Figure 5A). Sox9 was purified by His-bind resin chromatography. Because it contained an additional His Tag, the Mw values of the purified Sox9 was larger than its theoretical 53 kDa, which was approximately consistent with the SDS-PAGE result (Figure 5B).

### Oct4 Interacts With Sox9

**Figure 6:**
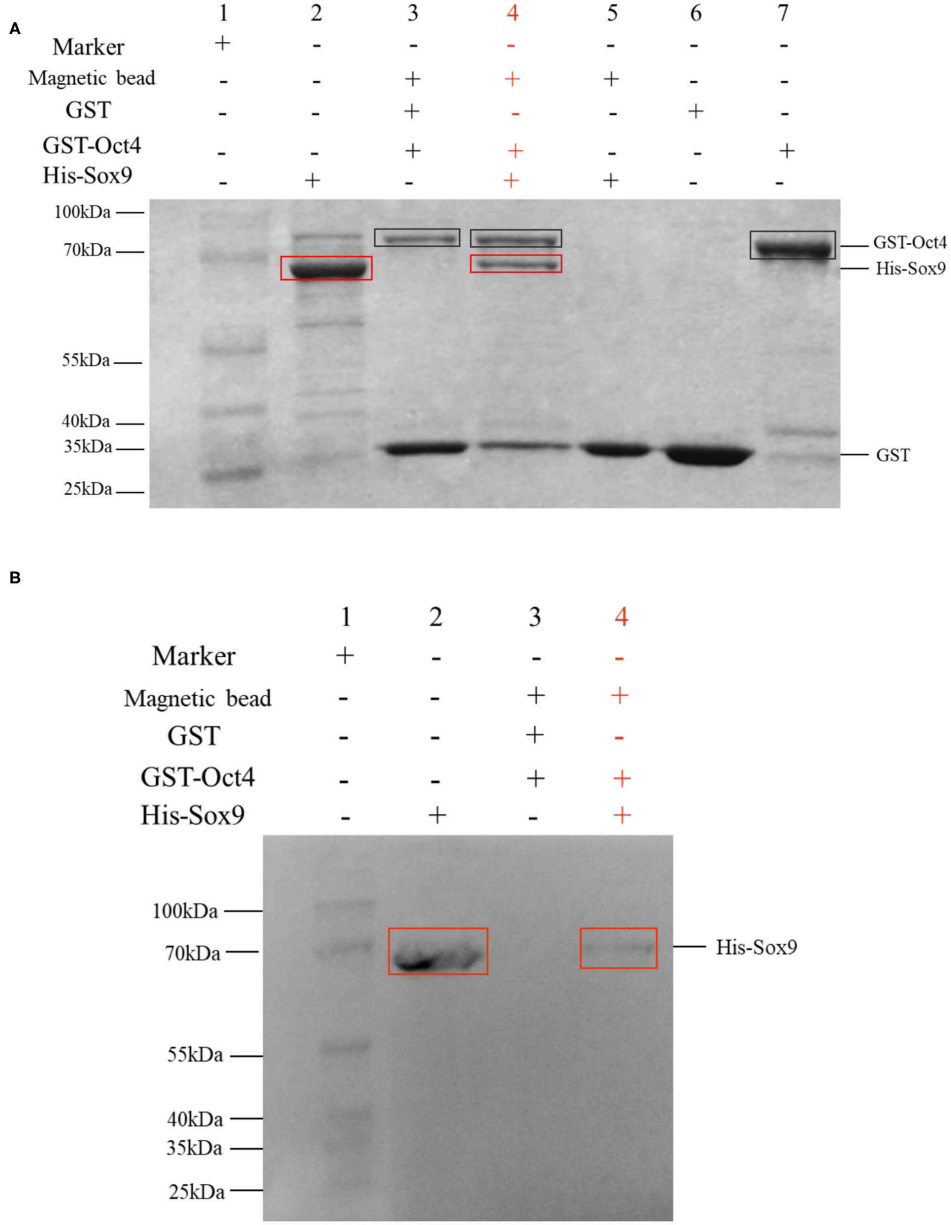
GST pull-down assay to determine the interaction between Oct4 and Sox9. Recombinant His-Sox9 was incubated with recombinant GST-Oct4. **(A)** Western blotting was performed using antibodies against His tags. **(B)** SDS-PAGE detection. The experimental group was shown in red. In the figures, the GST-Oct4 protein was marked with a black box, and His-Sox9 protein was marked with a red box.

The binding activity of Oct4 with Sox9 was examined by a pull-down assay. The results are shown in Figure 6A. In the SDS-PAGE electrophoresis detection, lane 3 is the control group with GST instead of pET30a-Sox9, and only two protein bands are GST and GST-Oct4; and the lane 4 is the experimental group with pET30a-Sox9, three protein bands are GST, GST-Oct4, and His-Sox9. GST-Oct4 and His-Sox9 were detected by anti-his polyclonal antibody in Western blot. The eluent showed that bands of the same size as His-Sox9 were detected in the experimental group in which pET30a-Sox9 was added (Figure 6B). Both results illustrated that pGEX-4T-Oct4 can interact with pET30a-Sox9 *in vitro*, while the negative control GST cannot bind to pET30a-Sox9.

## Discussion

In this study, the interaction between the two selected transcription factors and the critical promoter region of *SpVih* was verified by the EMSA experiment. EMSA is a common method for studying transcription factors *in vitro*, which can be used to detect the interaction of DNA and protein sequences. Through EMSA experiment, Li et al. (30) confirmed that Sox9 could directly bind to the COL10A1 gene promoter and activate its transcription, and Card et al. (31) found that Oct4 and Sox2 bind to a conserved promoter region of miR-302, a cluster of eight microRNAs expressed specifically in ESCs and pluripotent cells. In this experiment, a specific binding band can form when eyestalk nucleoprotein combined with the *Sp*TF-wt probe. However, the binding band became shallow when it combined with the 10-fold cold probe. At the same time, the binding band completely disappeared when the 100-fold cold probe was added, indicating that the cold probe can compete with the *Sp*TF-wt probe to bind certain components of eyestalk nucleoprotein. When 100-fold of *Sp*TF-mut probe (Oct4/Sox9 binding site was mutated) was added to the experimental system containing *Sp*TF-wt, the *Sp*TF-mut does not affect the formation of binding band, indicating that *Sp*TF-mut does not participate in the competition and proving that the formation of binding band is dependent on the presence of Oct4/Sox9 site. In the EMSA supershift experiment, when Oct4 or Sox9 antibody was added, the supershift band (DNA-Antigen-Antibody complex) appeared, indicating that Oct4 and Sox9 can directly and specifically bind to the key promoter region of *SpVih*.

In order to understand how Oct4 and Sox9 regulate *SpVih* expression, we constructed Oct4 and Sox9 overexpressed plasmids and *Sp*Vih core promoter fragment reporter vector. After they were co-transfected into HEK293T cell line, it was confirmed by double luciferase reporter gene that both transcription factors can promote *SpVih* expression and have a concentration effect. To verify the authenticity of the cell experiment results, we obtained Oct4 and Sox9 dsRNA by *in vitro* transcription. Then we injected the dsRNA into the eyestalks to interfere with the expression of two transcription factors in the crabs. The results showed that both Oct4 and Sox9 in the two experimental groups were successfully inhibited compared with the control group. The expression level of *SpVih* in the eyestalk decreased accordingly. Both the cell overexpression experiment and the *in vivo* RNA interference experiment showed that the transcription factors Oct4 and Sox9 could positively regulate *SpVih*.

Vitellogenin (VTG) is a precursor of vitellin and the main component of egg yolk proteins in oviparous animals. In the mud crab, it mainly exists in the gonads and hepatopancreas and can promote ovarian development and maturation (32). VIH can play a negative regulatory role in the expression of VTG (33). In the *S. olivacea*, VIH in the eyestalk is involved in inhibiting the production of VTG (34). Type II VIH in *P. vannamei* has the activity of inhibiting its vitellogenesis, and it can effectively inhibit the expression of Vtg mRNA in the hepatopancreas of the shrimp (35). In order to further explore the influence of Oct4 and Sox9 on *SpVtg*, we tested the expression level of *SpVtg* in ovary and hepatopancreas of mud crab after Oct4 and Sox9 interference. It was found that compared with the control group, as the *SpVih* expression level of the eyestalk in the RNA interference group decreased, the *SpVtg* expression level in the ovary or hepatopancreas increased significantly.

As transcription factors, Oct4 and Sox9 often interact with other transcription factors to achieve regulatory functions. Oct4 and Sox2 directly regulate the expression of another pluripotent transcription factor Zfp206 in embryonic stem cells (36). In Xenopus, Oct4 homolog Oct91 can bind to each type of SoxB1 (Sox1, Sox2, Sox3) protein and had different effects, Oct91 cooperates with Sox2 to maintain neural progenitor marker expression (37). During chondrogenesis, the direct binding of Sox9 and ap-1 promotes the activity of multiple genes, for example, the interaction of Sox9 and AP-1 synergistic activates the Col10a1 enhancer (38). Masuda and Esumi (39) found that Sox9 can regulate the expression of BEST1 in retinal pigment epithelium by interacting with the microphthalmia-associated transcription factors MITF and OTX2. In the cell transfection experiment, a group that simultaneously transfected two transcription factors with *SpVih* into the cell was set up, and their activity of *SpVih* was significantly enhanced compared to the group with only one transcription factor. It is speculated that there may be some interaction between the two transcription factors to strengthen the regulation of *SpVih*. In order to verify whether there is a real interaction between the two transcription factors, we obtained the Oct4 and Sox9 recombinant proteins with the GST tag and His tag through prokaryotic expression, respectively. GST pull-down analysis showed that there was indeed a direct interaction between Oct4 and Sox9.

In conclusion, the transcriptional regulation of *SpVih* by the transcription factors Oct4 and Sox9 in mud crab was further elaborated through cell overexpression, *in vivo* RNA interference, and GST pull-down. These results will help us to better understand the regulatory mechanism of *SpVih* gene, and therefore we hope that it will be possible to achieve the goal of promoting the development and maturity of crab ovaries by controlling key factors and finally apply the research results to production practice in the future.

## Data Availability Statement

The original contributions presented in the study are included in the article/supplementary material, further inquiries can be directed to the corresponding author/s.

## Ethics Statement

The animal study was reviewed and approved by Animal Ethics Committee of Jimei University.

## Author Contributions

JL, ZZh, and YW: conceptualization and writing—review and editing. JL, XJ, ZZo, and KL: methodology, investigation, and visualization. JL: software, validation, formal analysis, data curation, and writing—original draft. YW: resources, supervision, and project administration. YW and ZZh: funding acquisition. All authors contributed to the article and approved the submitted version.

## Conflict of Interest

The authors declare that the research was conducted in the absence of any commercial or financial relationships that could be construed as a potential conflict of interest.
